# Impact of thrombolytic therapy on basilar artery occlusion patients with atrial fibrillation: results from a multi-center prospective cohort study

**DOI:** 10.3389/fneur.2025.1634708

**Published:** 2025-07-16

**Authors:** Xianjin Shang, Longsheng Chu, Heming Chen, Ke Yang, Qian Yang, Wei Hu, Jie Xu, Zhiming Zhou

**Affiliations:** ^1^Department of Neurology, The First Affiliated Hospital of Wannan Medical College (Yijishan Hospital), Wuhu, Anhui, China; ^2^Department of Neurology, The First Affiliated Hospital of USTC, Division of Life Science and Medicine, University of Science and Technology of China, Hefei, Anhui, China; ^3^Institute of Brain Science, Wannan Medical College, Wuhu, Anhui, China; ^4^Department of Neurology, Beijing Tiantan Hospital, Capital Medical University, Beijing, China

**Keywords:** basilar artery occlusion (BAO), atrial fibrillation, intravenous thrombolysis, endovascular thrombectomy, bridging therapy

## Abstract

**Background:**

The benefits and risks of intravenous thrombolysis combined with endovascular treatment for basilar artery occlusion patients with atrial fibrillation (AF) are uncertain. This research investigates the disparities in the impact of bridging thrombolysis on the long-term prognoses of endovascular treatment between patients with AF and those without it.

**Methods:**

We analyzed data from a Chinese multi-center prospective registry conducted between March 2017 and February 2023. Primary analysis included favorable (mRS 0–2) and good (mRS 0–3) outcomes at 3 months, the risk ratio (aOR) and 95% confidence interval for the outcome associated with bridging thrombolysis were calculated using multivariate regression analysis. Subgroup analyses evaluated the relative excess risk index (REPI) for AF and intravenous thrombolysis.

**Results:**

Among 1,368 patients, the ratio of AF to non-AF patients was 434:934, the proportion receiving intravenous thrombolysis was 101 vs. 226. In the AF group, thrombolysis improved functional prognosis (aOR 1.93, 95% CI 1.14 to 3.29, *p* = 0.01 for favorable; aOR 2.06, 95% CI 1.23 to 3.46, *p* = 0.006 for good outcomes), with no differences in the non-AF group. Cross-stratification analysis indicated that AF patients receiving thrombolysis had the highest rates of favorable (aOR 1.99, 95% CI 1.27 to 3.11, *p* = 0.002) and good outcomes (aOR 2.11, 95% CI 1.35 to 3.30, *p* = 0.001), suggesting a significant additive effect of the treatments (REPI 0.89, 95% CI: 0.07–1.71; *p* = 0.02 and REPI 1.13, 95% CI: 0.24–2.02; *p* = 0.004).

**Conclusion:**

The presence of AF modified the treatment effect of bridging thrombolysis in basilar artery occlusion. These findings warrant confirmation through RCT studies.

## Introduction

Basilar artery occlusion (BAO) is a severe form of ischemic stroke. Recent clinical trials, ATTENTION (Endovascular Treatment for acute Basilar Artery Occlusion) and BAOCHE (Basilar Artery Occlusion Chinese Endovascular Trial), have confirmed that aggressive thrombectomy treatment can improve clinical functional outcomes for these patients. However, unlike the relatively favorable outcomes in the anterior circulation, patients undergoing thrombectomy for BAO still face high rates of mortality and disability (1–3).

Several clinical studies on acute large vessel occlusion in the anterior circulation have not consistently demonstrated that direct thrombectomy is superior to bridging therapy (4–9). However, in the atrial fibrillation (AF) subgroup, bridging therapy has been associated with better functional outcomes and lower mortality rates, suggesting that individualized AF risk factors might play a role in determining the optimal reperfusion strategy (10).

As for the benefits and risks of bridging therapy in posterior circulation, several studies and meta-analyses have yielded inconclusive results (11–17). The proportion of cardiogenic basilar artery embolism due to AF is relatively high in both Eastern and Western populations, ranging from approximately 21.0 to 32.6%. The benefit of endovascular treatment (EVT) in AF-related basilar artery embolism patients remains uncertain, as various studies report inconsistent outcomes (18–21). Whether intravenous thrombolysis (IVT) in combination with thrombectomy can improve clinical outcomes in AF patients is still unknown. Therefore, this study aims to investigate differences in EVT outcomes in AF and non-AF BAO patients undergoing thrombolytic therapy.

## Methods

### Patient criteria

This retrospective analysis included patients from a multi-center, nationwide, prospective registry study, ATTENTION (Endovascular Treatment for acute Basilar Artery Occlusion), which aimed to evaluate the efficacy and safety of EVT compared to medical therapy within 24 h of symptom onset in patients with acute BAO stroke in a real-world setting (http://www.chictr.org.cn; ChiCTR2000041117). This study inclusion criteria were as follows: (i) Patients with confirmed BAO via preoperative imaging (CTA/MRA/DSA) within 24 h of symptom onset. (ii) Pre-stroke modified Rankin Scale (mRS) score ≤2. (iii) Patients who consented to receive EVT and agreed to follow-up after discharge or via telephone. Exclusion criteria were: (i) Presence of intracranial hemorrhage detected before EVT. (ii) Missing research data for study patients. (iii) Currently undergoing anticoagulation treatment or at significant risk of severe bleeding; (iv) patients with systemic or terminal illnesses likely to lead to serious adverse outcomes in the short term.

Written informed consent forms were signed by all included patients and/or their legal representatives.

### Treatment protocol

Patients presenting within 4.5 h of symptom onset are informed of their eligibility for both thrombolysis and thrombectomy, in accordance with guideline recommendations. Intravenous thrombolysis (alteplase or tenecteplase) is administered after patients’ or their legal representative’s consent, if not, the patients cannot receive IVT and have further assessment. When there is concomitant large vessel occlusion, endovascular therapy is conducted after further communication (“bridging IVT” group). For patients presenting beyond 4.5 h, endovascular therapy is directly pursued after obtaining informed consent (“no bridging IVT” or direct thrombectomy group).

The recombinant alteplase dosage was set at 0.9 mg/kg of body weight, with the initial 10% given as a rapid intravenous push over 1 min, followed by the remaining 90% infused over the next hour. Tenecteplase is administered as a bolus injection at a dose of 0.25 mg/kg body weight.

Recanalization of the BAO was performed using devices approved for using in China, including direct aspiration thrombectomy, stent retrievers, balloon angioplasty, and stent placement, or a combination of these methods, as described previously (22). The specific technique employed was determined by interventional neuroradiologists with interventional expertise, adapting to the individual circumstances of each patient.

### Data collection

The patients’ data included demographic information (age, sex, pre-stroke mRS score), history of vascular risk factors (AF, hypertension, diabetes, hyperlipidemia, prior stroke or TIA), medication history (use of anticoagulants or antiplatelet agents), and ASPECTS scores based on preoperative conventional CT (with scores ranging from 0 to 10, where 0 indicates the highest infarct burden and 10 indicates normal imaging). Other collected data included the NIHSS score upon admission, onset time, treatment puncture time, and recanalization or endpoint time. The onset time primarily refers to when the patient was first observed to present symptoms; if the exact onset time is unclear, it is defined as the time since the patient was last seen normal, including wake-up but not unwitnessed stroke patients. AF was defined as a clear diagnosis before onset or documented during emergency treatment or endovascular therapy, characterized by transient or persistent episodes observed on electrocardiograms, Holter monitoring, or continuous cardiac monitoring. Oral anticoagulation therapy was indicated by either guardian reports or documented prescriptions for oral vitamin K antagonists (such as warfarin), novel oral anticoagulants, or physician’s prescription for intravenous heparin or low molecular weight heparin.

The distribution of the BAO was categorized into three segments based on the anterior inferior cerebellar artery and superior cerebellar artery, dividing the basilar artery into proximal, middle, and distal segments in relation to the heart. Accurate localization was determined through a combination of preoperative imaging assessments and intraoperative DSA angiography. The etiology of the BAO was classified according to the TOAST criteria, which assessed presumed stroke causative mechanisms. All assessments were conducted following standardized training, ensuring good inter-center agreement, with any discrepancies resolved through consensus.

### Outcome assessment

The standard for assessing recanalization in patients was based on the modified TICI score (mTICI), where a score of 0 indicates no antegrade flow, and a score of 3 indicates normal flow restoration. Recanalization was determined by binary classification based on the final angiography images, with scores of 0–2a defined as unsuccessful recanalization and scores of 2b-3 indicating successful recanalization. A follow-up cranial CT scan was routinely performed within 72 h postoperatively to check for hemorrhage. If a patient experienced a change in NIHSS score exceeding 4 points postoperatively, symptomatic intracranial hemorrhage was determined based on the ECASS-3 criteria; otherwise, it was classified as asymptomatic. At 90 days, follow-up was conducted through telephone interviews or outpatient visits to assess the patients’ modified Rankin Scale (mRS) scores (where 0 indicates full recovery and 6 indicates death). The follow-up assessments were carried out by independent, experienced assessors who were unaware of the specific treatments the patients received. An mRS score of 0–3 was defined as good outcome, and mRS 0–2 were defined as favorable outcome.

### Statistical analysis

The normality of continuous data was assessed using the Kolmogorov–Smirnov test. Continuous data that followed a normal distribution were analyzed using the Student’s *t*-test, while non-normally distributed continuous data were analyzed using the Mann–Whitney *U* test. Categorical data comparisons were conducted using the chi-square test or Fisher’s exact test. A multivariable logistic regression model was employed to adjust for variables that could potentially influence outcomes after EVT based on previously known factors, including age, sex, history of vascular risk factors (hypertension, diabetes, hyperlipidemia), bridging IVT, initial NIHSS score, posterior circulation ASPECTS, collateral scores, and time from onset to puncture. The results were expressed as odds ratios (OR) with 95% confidence intervals (CI) and *p* values. Exploratory subgroup analyses were performed to evaluate the potential modifying effect of AF on outcomes, based on whether patients had AF in the context of BAO. Patients were stratified into four groups according to the presence of AF and prior thrombolytic treatment. The additive or multiplicative effects of these factors were assessed by calculating the relative excess risk due to interaction (RERI) and adjusted OR (aOR) with 95% CI and *p* values to analyze the combined modifying effects. A two-tailed p value of less than 0.05 was considered statistically significant. All data analyses were conducted using SPSS software 23.0 (SPSS Inc., Chicago, IL) and R studio 3.6.0 statistical packages.

## Results

**Figure 1:**
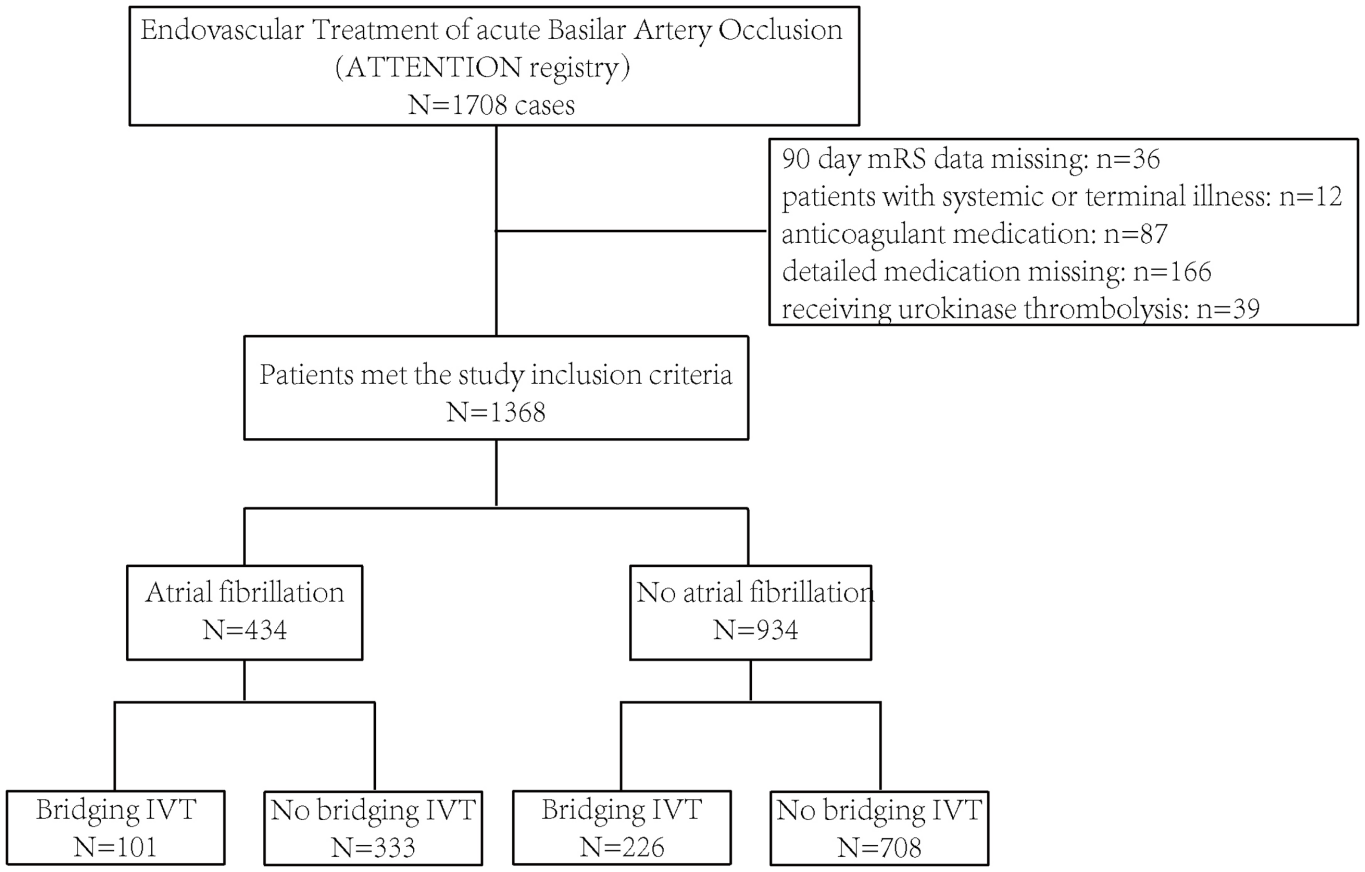
Study flow chart.

A total of 1,368 patients with BAO were included, excluding those with an unclear anticoagulation history (Figure 1). The mean age was 64.5 ± 11.9 years, and 31.3% were female. Patients were divided into four groups based on a history of AF and bridging thrombolysis. Among the 434 patients with AF, 101 (23.3%) received thrombolytic treatment before the intervention, while 226 of the remaining 934 patients (24.2%) underwent bridging IVT (Table 1).

**Table 1:** Basic characteristics of posterior circulation acute ischemic stroke patients who underwent endovascular thrombectomy (*n* = 1368).

Variable	Atrial fibrillation (*n* = 434)			No atrial fibrillation (*n* = 934)		
	Bridging IVT (*n* = 101)	No bridging IVT (*n* = 333)	*P* value	Bridging IVT (*n* = 226)	No bridging IVT (*n* = 708)	*P* value
Demographic information						
Age (yrs), median (IQR)	63 (52.5–69)	67 (58–74.5)	<0.001	66 (56–73)	56 (65–73)	0.70
Male, number (%)	68 (67.3)	221 (66.4)	0.85	166 (73.5)	485 (68.5)	0.18
Vascular risks						
Hypertension, number (%)	64 (63.4)	219 (65.8)	0.65	158 (69.9)	477 (67.4)	0.51
Diabetes mellitus, number (%)	21 (20.8)	85 (25.5)	0.33	69 (30.5)	187 (26.4)	0.23
Dyslipidemia, number (%)	40 (39.6)	88 (26.4)	0.01	89 (39.4)	177 (25.0)	<0.001
Previous stroke/TIA, number (%)	18 (17.8)	68 (20.4)	0.56	42 (18.6)	124 (17.5)	0.76
Admission data						
Onset NIHSS, median (IQR)	20 (12.5–29)	22 (14–32)	0.34	19 (11–30)	20 (12–29)	0.40
Pc-ASPECTS, median (IQR)	9 (8–10)	8 (8–10)	0.16	9 (8–10)	8 (7–10)	0.43
Collateral assessment, median (IQR)	0 (0–1)	0 (0–1)	0.35	0 (0–2)	0 (0–1)	0.46
TOAST classification			0.43			0.06
Large artery atherosclerosis, number (%)	0 (0)	0 (0)		128 (56.6)	434 (61.3)	
Cardioembolic, number (%)	101 (100.0)	333 (100.0)		44 (19.5)	93 (13.1)	
Others/undetermined, number (%)	0 (0)	0 (0)		54 (23.9)	181 (125.6)	
Radiographic presentation						
Location of occlusion, number (%)			0.09			0.15
Proximal segment	31 (30.7)	77 (23.1)		96 (42.5)	250 (35.3)	
Middle segment	28 (27.7)	130 (39.0)		61 (27.0)	216 (30.5)	
Distal segment	42 (41.6)	126 (37.8)		69 (30.5)	242 (34.2)	
Procedural data						
Time from onset to puncture (min), median (IQR)	344 (311–387.5)	544 (343.5–764.5)	<0.001	342 (275–374.5)	517.5 (275–751)	<0.001
Time from onset to reperfusion (min), median (IQR)	435 (389.5–496)	633 (423–852)	<0.001	429.5 (379.7–482.5)	609 (401.2–849)	<0.001
Time from puncture to reperfusion (min), median (IQR)	84 (64–105)	89 (68–108)	0.15	85 (65–112)	89 (69–110)	0.29

IVT, intravenous thrombolysis; IQR, interquartile range; TIA, transient ischemic attack.

In the overall cohort of patients, AF patients had more severe initial neurological symptoms (21 vs. 20, *p* = 0.02) compared to non-AF patients. Significant differences of the causes and vascular occlusion sites were also observed between the two groups (both *p* < 0.001).

When comparing AF patients with and without bridging IVT, those receiving IVT were younger (63 vs. 67 years, *p* < 0.001) and had higher rates of hyperlipidemia (39.6% vs. 26.4%, *p* = 0.01). They also had shorter times from symptom onset to puncture (344 vs. 544 min, *p* < 0.001) and to reperfusion (435 vs. 633 min, *p* < 0.001).

For non-AF patients, those who received IVT had a higher prevalence of hyperlipidemia (39.4% vs. 25%, *p* < 0.001) and shorter times from onset to puncture (342 vs. 517.5 min, *p* < 0.001) and from onset to reperfusion (429.5 vs. 609 min, *p* < 0.001). Other baseline characteristics were comparable between groups.

### Primary outcome

In the overall cohort, bridging IVT was not significantly linked to favorable outcomes at 90 days (aOR 1.19, 95% CI 0.89 to 1.60, *p* = 0.22). However, among patients with AF, those treated with bridging IVT had a higher rate of favorable outcomes compared to those receiving EVT alone (47.5% vs. 30.3%, *p* = 0.001), and good outcome (57.4% vs. 37.2%, *p* < 0.001). Conversely, non-AF patients exhibited similar outcomes regardless of bridging IVT (favorable: 34.1% vs. 33.3%, *p* = 0.83; good: 39.4% vs. 40.1%, *p* = 0.84) (Table 2).

**Table 2:** Outcomes of posterior circulation acute ischemic stroke patients who underwent endovascular thrombectomy with and without atrial fibrillation stratified by bridging intravenous thrombolysis (*n* = 1368).

Variable	Atrial fibrillation (*n* = 434)			No atrial fibrillation (*n* = 934)		
	Bridging IVT (*n* = 101)	No bridging IVT (*n* = 333)	*P* value	Bridging IVT (*n* = 226)	No bridging IVT (*n* = 708)	*P* value
mRS0-2, number (%)	48 (47.5)	101 (30.3)	0.001	77 (34.1)	236 (33.3)	0.83
mRS0-3, number (%)	58 (57.4)	124 (37.2)	<0.001	89 (39.4)	284 (40.1)	0.84
Successful reperfusion (mTICI ≥2b), number (%)	84 (83.2)	294 (88.3)	0.17	190 (84.1)	626 (88.4)	0.08
Symptomatic ICH, number (%)	6 (5.9)	16 (4.8)	0.64	8 (3.5)	33 (4.7)	0.47
Asymptomatic ICH, number (%)	4 (4.0)	8 (2.4)	0.40	7 (3.1)	23 (3.2)	0.91

ICH, intracranial hemorrhage; IVT, intravenous thrombolysis; mRS, modified Rankin Score; mTICI, modified Thrombolysis in Cerebral Infarction.

Exploratory subgroup analysis indicated that AF patients receiving bridging IVT had the highest odds of favorable outcomes (aOR 1.99, 95% CI 1.27 to 3.11, *p* = 0.002) compared to non-AF patients without bridging IVT, followed by non-AF patients with bridging IVT (aOR 0.98, 95% CI 0.70 to 1.37, *p* = 0.91), AF patients without bridging IVT (aOR 0.94, 95% CI 0.70 to 1.27, *p* = 0.70).

Within the AF subgroup, bridging IVT was significantly associated with favorable outcomes (aOR 1.93, 95% CI 1.14 to 3.29, *p* = 0.01), but this was not observed in the non-AF cohort (aOR 0.91, 95% CI 0.63 to 1.30, *p* = 0.62). Additionally, the presence of AF modified the relationship between bridging IVT and favorable outcomes, evident in both the multiplicative (ratio of ORs 2.02, 95% CI 1.12 to 3.66, *p* < 0.05) and additive (RERI 0.89, 95% CI 0.07 to 1.71, *p* = 0.02) scales (Table 3).

**Table 3:** Modification of the effect of bridging thrombolysis on favorable outcomes in posterior circulation acute ischemic stroke patients that underwent endovascular thrombectomy by presence of concomitant atrial fibrillation.

Variables	No bridging IVT		Bridging IVT		aOR (95%CI) for bridging IVT within strata of AF status
	*N* with/without favorable outcomes	aOR (95%CI)	*N* with/without favorable outcomes	aOR (95%CI)	
No AF	236/472	1.0	77/149	0.982 (0.700–1.376); *p* = 0.915	0.914 (0.639–1.308);*p* = 0.624
AF	101/232	0.944 (0.701–1.272);*p* = 0.706	48/53	1.994 (1.279–3.110);p = 0.002	1.939 (1.143–3.291);*p* = 0.014

Measure of effect modification on addictive scale: RERI 0.89, 95%CI: 0.07–1.71, *p* = 0.02.

Measure of effect modification on multiplicative scale: ratio of ORs 2.02, 95%CI: 1.12–3.66.

AF, atrial fibrillation; aOR, adjusted odd ratio; IVT, intravenous thrombolysis.

Adjusted variables including age, sex, history of vascular risk factors (hypertension, diabetes, hyperlipidemia), bridging IVT, initial NIHSS score, posterior circulation ASPECTS, collateral scores, and time from onset to puncture.

Exploratory subgroup-stratified analysis showed that AF patients receiving bridging IVT had the highest odds of achieving good outcomes at 90 days (aOR 2.11, 95% CI 1.35 to 3.30, *p* = 0.001) compared to non-AF patients without bridging IVT, followed by AF patients without bridging IVT (aOR 0.98, 95%CI 0.73 to 1.30, *p* = 0.89), non-AF patients with bridging IVT (aOR 0.93, 95% CI 0.67 to 1.29, *p* = 0.69). Among AF patients, bridging IVT was significantly associated with good outcomes (aOR 2.06, 95% CI 1.23 to 3.46, *p* = 0.006), whereas no such association was found in the non-AF group (aOR 0.89, 95% CI 0.63 to 1.26, *p* = 0.53). The presence of AF modified the relationship between bridging IVT and good outcomes, confirmed by both the multiplicative scale (ratio of ORs 2.33, 95%CI: 1.31–4.17, *p* < 0.05) and the additive scale (RERI 1.13, 95%CI: 0.24–2.02, *p* = 0.004) (Table 4).

**Table 4:** Modification of the effect of bridging thrombolysis on good outcome in posterior circulation acute ischemic stroke patients that underwent endovascular thrombectomy by presence of concomitant atrial fibrillation.

Variables	No bridging IVT		Bridging IVT		aOR (95%CI) for bridging IVT within strata of AF status
	*N* with/without good outcomes	aOR (95%CI)	*N* with/without good outcomes	aOR (95%CI)	
No AF	284/424	1.0	89/137	0.937 (0.678–1.297);*p* = 0.697	0.897 (0.635–1.267); *p* = 0.538
AF	124/209	0.982 (0.739–1.303); *p* = 0.898	58/43	2.118 (1.358–3.301);p = 0.001	2.063 (1.230–3.460); *p* = 0.006

Measure of effect modification on addictive scale: RERI 1.13, 95%CI: 0.24–2.02, *p* = 0.004.

Measure of effect modification on multiplicative scale: ratio of ORs 2.33, 95%CI: 1.31–4.17.

AF, atrial fibrillation; aOR, adjusted odd ratio; IVT, intravenous thrombolysis.

Adjusted variables including age, sex, history of vascular risk factors (hypertension, diabetes, hyperlipidemia), bridging IVT, initial NIHSS score, posterior circulation ASPECTS, collateral scores, and time from onset to puncture.

### Secondary outcomes

**Figure 2:**
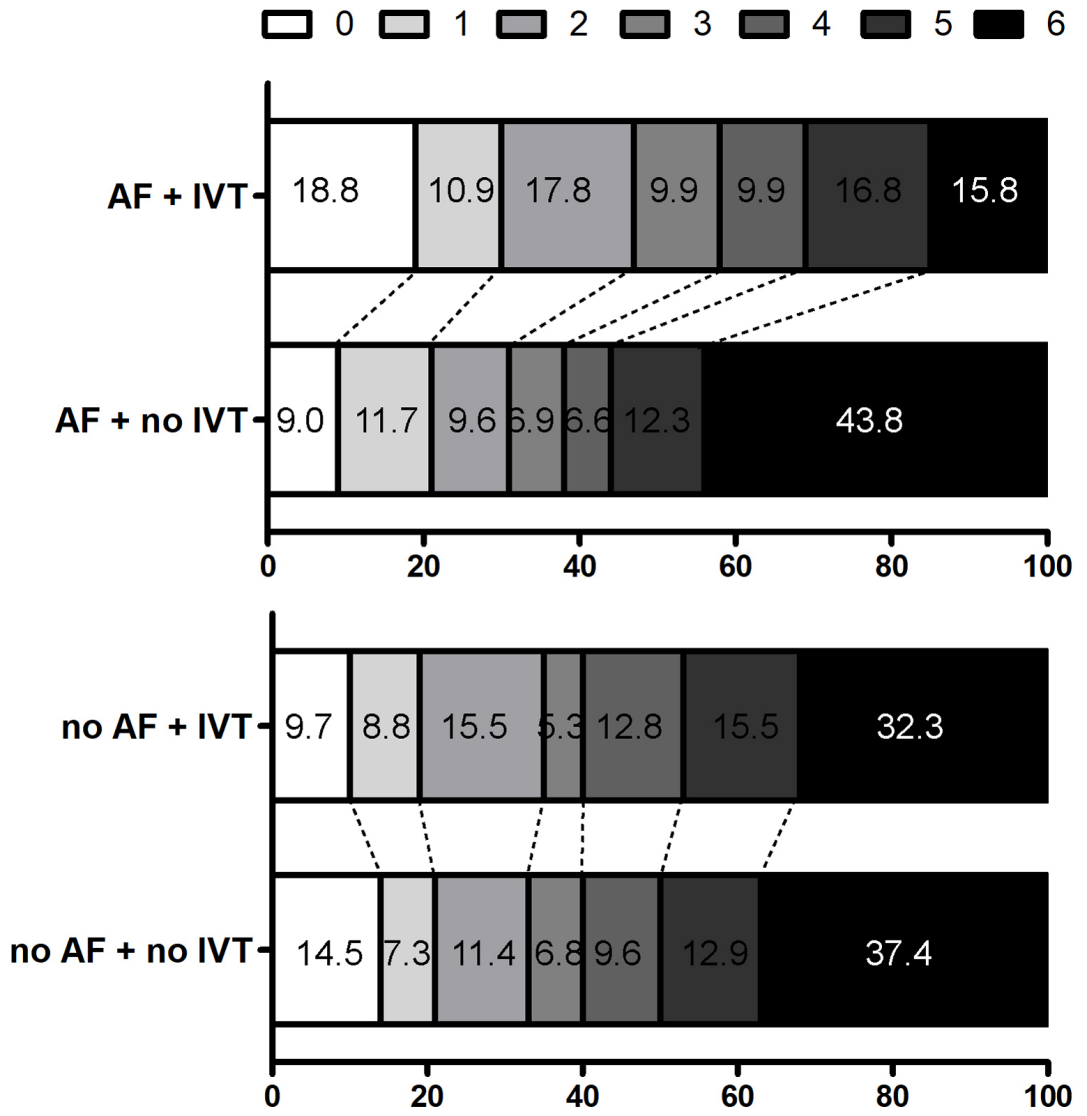
Distribution of the mRS scores at 90 days in patients. AF, atrial fabrillation; IVT, intravenous thrombolysis.

Successful reperfusion (mTICI ≥ 2b) and rates of non-symptomatic or symptomatic intracranial hemorrhage were similar across groups, regardless of AF presence or bridging IVT exposure. Among AF patients, 83.2% with bridging IVT achieved successful reperfusion compared to 88.3% in the direct EVT group (*p* = 0.17). Symptomatic intracranial hemorrhage rate was 5.9% in the bridging IVT group and 4.8% in the direct EVT group (*p* = 0.64). Notably, the 90-day mortality rate was significantly lower in the bridging IVT group (15.8% vs. 43.8%, *p* < 0.001) (Table 2, Figure 2).

## Discussion

This study examined the combined effects of AF and bridging thrombolysis on the outcomes of EVT in BAO patients, which showed bridging thrombolysis had better functional outcomes, lower 90-day mortality without increasing bleeding risks in the AF cohort.

Approximately 20% of stroke patients have atrial fibrillation (AF) as a risk factor, and more than 40% of large vessel occlusion strokes are associated with AF (23). AF often leads to sudden symptoms and proximal vessel occlusions, resulting in greater ischemic damage and increasing bleeding risks, making timely reperfusion critical for patient prognosis (24–26). However, AF-related BAO often resulted in futile recanalization due to inadequate collateral circulation. AF remains a poor prognostic factor for patients with BAO (20, 27). Our findings corroborate other research indicating that clinical outcomes for thrombectomy do not significantly differ based on AF presence (19, 21). Thus, prognosis differences among different treatment groups may be more closely related to whether IVT was administered.

Just as previous studies on anterior circulation infarction have demonstrated that bridging therapy is not always inferior to direct thrombectomy, the favorable functional outcomes resulting from bridging therapy also vary among patients with infarction related to atrial fibrillation or not (4–9, 28). However, these results mainly pertain to anterior circulation strokes. Some research on BAO has shown no significant differences in outcomes between bridging thrombolysis and thrombectomy versus thrombectomy alone (13, 17). But other studies suggest bridging treatment may improve outcomes, Nappini et al. (15) noting the effectiveness primarily within 6 h of symptom onset in BAO patients (16). Siow’s research also implies that patients with atherosclerosis may benefit more from bridging thrombolysis (17). But, the suitability of early thrombolysis for BAO specifically caused by AF-related embolism remains underexplored.

Patients with AF receiving bridging therapy have an increased bleeding risk without clear improvement in functional outcomes (29), but it did not differentiate between anterior and posterior circulation strokes, it cannot be definitively concluded that bridging therapy is harmful for patients with posterior circulation stroke. The posterior circulation’s anatomy, with a stronger collateral network and better ischemic tolerance, offers improved distal perfusion and more time for recanalization compared to the anterior circulation. Based on prior studies and this article, bridging thrombolysis may enhance embolus removal and perforator blood supply. The rich posterior collateral circulation may also aid retrograde basilar artery filling and improve thrombolysis efficacy, as seen in anterior circulation studies (30, 31). Whether there were better outcomes of thrombolytic therapy in AF patients are due to earlier thrombectomy treatment in bridging thrombolysis group, we conducted additional sensitivity analyses, and found AF patients who underwent bridging therapy within 6 h still had better clinical outcomes. The results further support initiating bridging therapy earlier in BAO patients.

This study has several limitations: First, this was a retrospective analysis from the prospective ATTENTION registry in China, potentially limited its generalizability. Second, the administration time of thrombolytic agents was not available, which was considered to have an important impact on prognosis, and different thrombolytic agents could yield varying effects, because tenecteplase agent showed higher recanalization rate before thrombectomy. Third, prior anticoagulant medication was a contraindication for IVT treatment, although the inclusion criteria excluded these patients in this study, recall bias from patients and family members cannot be entirely ruled out. Fourth, although all patients received standardized post-discharge medication guidance, data on medication adherence, care adequacy and complication management were unavailable. While these factors may affect outcomes, they are unlikely to exert major influence compared to acute reperfusion therapies. Finally, while our study population was restricted to patients with comorbid AF, this cohort represents a substantial subgroup in clinical practice. The findings could provide valuable insights to guide personalized therapeutic decision-making for these high-risk populations.

## Conclusion

The presence of atrial fibrillation (AF) influenced the treatment effect of bridging thrombolysis in BAO patients. In this patient group, bridging thrombolysis showed promise in enhancing clinical outcomes and lowering mortality risk, without causing an increase in procedural hemorrhagic complications. Therefore, bridging thrombolysis is likely to remain the standard treatment for acute BAO, particularly in patients with comorbid AF. However, these findings need to be validated through randomized controlled trials.

## Data Availability

The raw data supporting the conclusions of this article will be made available by the authors, without undue reservation.
