# Correction: Impact of thrombolytic therapy on basilar artery occlusion patients with atrial fibrillation: results from a multi-center prospective cohort study

**DOI:** 10.3389/fneur.2025.1670667

**Published:** 2025-09-03

**Authors:** Xianjin Shang, Longsheng Chu, Heming Chen, Ke Yang, Qian Yang, Wei Hu, Jie Xu, Zhiming Zhou

**Affiliations:** ^1^Department of Neurology, The First Affiliated Hospital of Wannan Medical College (Yijishan Hospital), Wuhu, Anhui, China; ^2^Department of Neurology, The First Affiliated Hospital of USTC, Division of Life Science and Medicine, University of Science and Technology of China, Hefei, Anhui, China; ^3^Institute of Brain Science, Wannan Medical College, Wuhu, Anhui, China; ^4^Department of Neurology, Beijing Tiantan Hospital, Capital Medical University, Beijing, China

**Keywords:** basilar artery occlusion (BAO), atrial fibrillation, intravenous thrombolysis, endovascular thrombectomy, bridging therapy

In the published article, Supplementary Tables 1 and 2 were erroneously published as Table 1 and Table 2. The supplementary tables have been added as Supplementary material. The correct version of Table 1 and Table 2 has been published in the original article.

The original version of this article has been updated.

