# Supplementary material for: Impact of thrombolytic therapy on basilar artery occlusion patients with atrial fibrillation: results from a multi-center prospective cohort study
Source: Front Neurol. 2025 Jul 16;16:1634708. doi: 10.3389/fneur.2025.1634708 (PMC12309002; doi:10.3389/fneur.2025.1634708)
Supplement: Supplementary file 1 [file Table_1.docx]

**Supplemental Table 1.** Basic characteristics and outcomes of posterior circulation acute ischemic stroke patients who underwent endovascular thrombectomy (n=1368)

| variables | Onset to treatment ≤360 min (n=512) | | | Onset to treatment >360 min (856) | | |
| --- | --- | --- | --- | --- | --- | --- |
|  | AF (n=154) | No AF(n=358) | P value | AF (n=280) | No AF(n=576) | P value |
| Demographic information |  |  |  |  |  |  |
| Age (yrs), mean (SD) | 64.5±11.4 | 65.5±11.1 | 0.34 | 64.9±11.9 | 63.7±12.5 | 0.15 |
| Male, number (%) | 111(72) | 253(71) | 0.74 | 178（63.6） | 398(69.1) | 0.12 |
| Vascular risks |  |  |  |  |  |  |
| Hypertension, number (%) | 101(66) | 260(73) | 0.11 | 182(65.0) | 375(65.1) | 0.99 |
| Diabetes mellitus, number (%) | 33(21) | 89(25) | 0.40 | 73(26.1) | 167(29.0) | 0.41 |
| Dyslipidemia, number (%) | 45(29) | 97(27) | 0.62 | 83(29.6) | 169(29.3) | 0.93 |
| Admission data |  |  |  |  |  |  |
| Onset NIHSS, median (IQR) | 21(13-30) | 19(11-29) | 0.21 | 22.5(13-32) | 20(12-29) | 0.06 |
| Pc-ASPECTS, median (IQR) | 9(8-10) | 9(8-10) | 0.11 | 8(7.0-10.0) | 8(7-10) | 0.89 |
| Location of occlusion, number (%) |  |  | 0.01 |  |  | <0.01 |
| Proximal segment | 41(26.6) | 142(39.7) |  | 67(23.9) | 204(35.4) |  |
| Middle segment | 48(31.2) | 102(28.5) |  | 110(39.3) | 175(30.4) |  |
| Distal segment | 65(42.2) | 114(31.8) |  | 103(36.8) | 197(34.2) |  |
| Procedural data |  |  |  |  |  |  |
| Time from puncture to reperfusion (min), median (IQR) | 88.5(67.8-107.8) | 89(67-109.5) | 0.78 | 88.0(66.0-107.0) | 89(69-112) | 0.27 |
| Successful reperfusion (mTICI ≥2b), number (%) | 133(86) | 314(88) | 0.67 | 245(87.5) | 502(87.2) | 0.91 |

ASPECTS, Alberta Stroke Program Early Score; ICH, intracerebral hemorrhage; IQR, Interquartile Range; IVT, intravenous thrombolysis; mTICI, modified Thrombolysis In Cerebral Infarction; NIHSS, National Institute of Health Stroke Scale; TOAST, Trial of ORG 10172 in Stroke Treatment.
