# Supplementary material for: Impact of thrombolytic therapy on basilar artery occlusion patients with atrial fibrillation: results from a multi-center prospective cohort study
Source: Front Neurol. 2025 Jul 16;16:1634708. doi: 10.3389/fneur.2025.1634708 (PMC12309002; doi:10.3389/fneur.2025.1634708)
Supplement: Supplementary file 2 [file Table_2.docx]

**Supplemental Table 2**. The effect of bridging thrombolysis on clinical outcome between AF and no AF patients stratified by the treatment time.

| variable | Onset to treatment ≤360 min (n=512) | | | Onset to treatment >360 min (n=856) | | |
| --- | --- | --- | --- | --- | --- | --- |
|  | AF (n=154) | No AF(n=358) | aOR(95% CI), P value | AF (n=280) | No AF(n=576) | aOR(95% CI), P value |
| Symptomatic ICH, number (%) | 6(4) | 19(5) | 0.85(0.36-2.0); P=0.72 | 16(5.7) | 22(3.8) | 0.87(0.32-2.37);P=0.78 |
| Asymptomatic ICH, number (%) | 9(6) | 13(4) | 0.95(0.38-2.36); P=0.92 | 3(1.1) | 17(3.0) | 1.24(0.34-4.55);P=0.73 |
| mRS0-2, number (%) | 53(34.4) | 118(33.0) | 1.64(1.07-2.51); P=0.02 | 96(34.3) | 195(33.9) | 0.93(0.58-1.48);P=0.75 |
| mRS0-3, number (%) | 69(44.8) | 134(37.4) | 1.64(1.09-2.47); P=0.01 | 113(40.4) | 239(41.5) | 0.93(0.60-1.45);P=0.75 |

Adjusted variables including age, sex, history of vascular risk factors (hypertension, diabetes, hyperlipidemia), bridging IVT, initial NIHSS score, pc-ASPECTS score, location of occlusion, collateral scores, and time from puncture to reperfusion.
